# PlasClass improves plasmid sequence classification

**DOI:** 10.1371/journal.pcbi.1007781

**Published:** 2020-04-03

**Authors:** David Pellow, Itzik Mizrahi, Ron Shamir

**Affiliations:** 1 Blavatnik School of Computer Science, Tel Aviv University, Tel Aviv, Israel; 2 Department of Life Sciences, Ben-Gurion University of the Negev and the National Institute for Biotechnology in the Negev, Marcus Family Campus, Beer-Sheva, Israel; Johns Hopkins University, UNITED STATES

## Abstract

Many bacteria contain plasmids, but separating between contigs that originate on the plasmid and those that are part of the bacterial genome can be difficult. This is especially true in metagenomic assembly, which yields many contigs of unknown origin. Existing tools for classifying sequences of plasmid origin give less reliable results for shorter sequences, are trained using a fraction of the known plasmids, and can be difficult to use in practice. We present PlasClass, a new plasmid classifier. It uses a set of standard classifiers trained on the most current set of known plasmid sequences for different sequence lengths. We tested PlasClass sequence classification on held-out data and simulations, as well as publicly available bacterial isolates and plasmidome samples and plasmids assembled from metagenomic samples. PlasClass outperforms the state-of-the-art plasmid classification tool on shorter sequences, which constitute the majority of assembly contigs, allowing it to achieve higher F1 scores in classifying sequences from a wide range of datasets. PlasClass also uses significantly less time and memory. PlasClass can be used to easily classify plasmid and bacterial genome sequences in metagenomic or isolate assemblies. It is available under the MIT license from: https://github.com/Shamir-Lab/PlasClass.

This is a *PLOS Computational Biology* Software paper.

## Introduction

When using high-throughput sequencing to study the presence and dynamics of plasmids in their bacterial hosts, it is often necessary to classify sequences as being of plasmid or chromosomal origin. This is especially true in the case of metagenomic sequencing, which can include many sequences of unknown origin and varying lengths. We focus on the challenge of classifying contigs in a metagenomic assembly in order to identify which are of plasmid origin.

The current state-of-the-art classifier of plasmid sequences is PlasFlow [1], a neural network based algorithm that was shown to perform better than previous tools such as cBar [2]. While PlasFlow is successful in classifying small sets of long sequences, it produces less reliable results for short sequences and requires large memory on very large metagenomic datasets.

Here we present PlasClass, a new plasmid sequence classifier implemented as an easy to use Python package. It uses a set of logistic regression classifiers each trained on sequences of a different length sampled from plasmid and bacterial genome reference sequences. When applied on a set of sequences, the appropriate length-specific classifier is used for each sequence.

We tested PlasClass on simulated data, on bacterial isolates, on a wastewater plasmidome, and on plasmids assembled from human gut microbiome samples. For shorter sequences, which are the majority of contigs in an assembly, PlasClass achieved better F1 scores than PlasFlow. This resulted in better overall performance across all the datasets tested. PlasClass also used significantly less RAM and disk memory than PlasFlow, and can be run much faster by using multiprocessing.

PlasClass is provided at https://github.com/Shamir-Lab/PlasClass.

## Design and implementation

### Training databases

We used reference sequence databases to obtain the training sequences for our classifiers. For the plasmid references we used plasmid sequences listed in PLSDB [3] (v.2018_12_05), an up-to-date curated plasmid database. After filtering out duplicate sequences this database contained 13469 reference plasmids (median length: 53.8kb).

For the bacterial chromosome references we downloaded all complete bacterial genome assemblies from NCBI (download date January 9, 2019). We removed sequences annotated as being plasmids and filtered out duplicates, leaving 13491 reference chromosomes (median length: 3.7Mbp).

One quarter of the sequences were randomly removed from the databases before training in order to provide a held-out test set for validation. PlasClass was retrained on the full databases and this version was used for testing on assembled data.

### Training the classifiers

We sampled sequence fragments of different lengths from the reference sequences with replacement and constructed a k-mer frequency vector for each fragment. Canonical k-mers of lengths 3–7 were used, resulting in a feature vector of length 10952 for each fragment. Fragment lengths were 500k, 100k, 10k, and 1k. For the two shorter lengths, 90,000 training fragments were used from each class. For the lengths 500k and 100k, since there were not enough long plasmids to do the same, we sampled enough fragments to cover all of the sufficiently long plasmids to a depth of 5. This resulted in 1934 and 45525 plasmid fragments of length 500k and 100k, respectively on the full plasmid database.

For each length, a logistic regression classifier was trained on the plasmid and chromosomal fragments’ k-mer frequency vectors using the scikit-learn [4] machine learning library in Python. Code is provided to retrain the models on user-supplied reference sequence databases.

### Length-specific classification

PlasClass uses four logistic regression models to classify sequences of different length. Each sequence is assigned to the closest length from among 1kb, 10kb, 100kb, and 500kb. Equivalently, this defines four length ranges: (0,5.5kb], (5.5kb,55kb], (55kb,300kb], (300kb, ∞). Given a sequence, its k-mers are counted, the canonical k-mer frequency vector is calculated and used to classify it with the classifier for the range it falls into. k-mer counting can be performed in parallel for different sequences. Finally, all classification results are concatenated into a single output in the same order as the input sequences.

### Classification with PlasClass

PlasClass is available at https://github.com/Shamir-Lab/PlasClass. It has been retrained using the full set of database references. PlasClass can be used as a command-line tool to classify sequences in an input fasta file or it can be imported as a module into the user’s code to classify sequences in the user’s program. It can be run in parallel mode to achieve faster runtimes. PlasClass is fully documented in S1 File and at the url provided above.

## Results

We tested performance of PlasClass on both simulated and real data and compared it to PlasFlow.

### Experimental settings

PlasClass and PlasFlow both assign class probabilities to each sequence. We say a sequence is classified as having plasmid origin if the probability that it belongs to the plasmid class is > 0.5. When running PlasFlow, this probability was summed over all plasmid classes, and we set the parameter --threshold = 0.5 to ensure each sequence is classified as either plasmid or bacterial. All assemblies were performed using the --meta option of SPAdes [5] v3.12.

### Performance metrics

We calculated the precision, recall and F1 scores counting the *number* of true positive and false positive predictions. Some previous works [1, 6] calculated performance based on the *lengths* of the sequences classified as plasmids and the total length of the plasmids in a sample. A length-weighted metric is appropriate in the context of plasmid sequence assembly, but in the context of contig classification this makes little sense. (Consider the extreme case of one extremely long sequence and 999 very short ones. Classifying the long contig is easy, but a classifier that only identifies it correctly will have weighted precision and recall near 1 even though only 1/1000 of the sequences are correctly classified.) For this reason we used the numbers of correctly classified sequences.

On the assembled contigs we follow the previous works [1, 6] and consider a contig to be from the plasmid class if it matches a plasmid reference sequence—even if it also matches a chromosomal reference sequence. This is appropriate for classifying all sequences in an assembly to determine their origin. However, when constructing a benchmark for a classifier, it may be more suitable to filter ambiguous sequences that may belong to both classes out of the test set. For this reason, we also report results with all ambiguous sequences filtered out in S2 File.

### Classifying sequences from held-out references

We sampled overlapping *L*-long fragments covering the held out plasmids with an overlap of *L*/2 for *L* = 100k, 10k and 1k. A matching number of *L*-long fragments were sampled from the held out bacterial genomes for each length *L*. (Note that this creates a balanced classification scenario.) Table 1 summarizes the classification results. PlasClass improved precision at the cost of slightly lower recall and had better overall F1 on the shorter sequence lengths. These short sequences can make up the majority of contigs in metagenomic assemblies, allowing PlasClass to outperform PlasFlow in many settings as shown below.

**Table 1:** Performance on held out data.

Length (bp)	# fragments per class	PlasClass			PlasFlow		
		Precision	Recall	F1	Precision	Recall	F1
100k	2979	96.9	85.4	90.8	95.6	88.4	91.9
10k	56583	88.7	86.4	87.6	83.1	87.7	85.3
1k	607656	75.1	74.6	74.8	59.7	79.1	68.1

Performance of PlasClass and PlasFlow on fixed length sequence fragments sampled from the held out references.

### Performance on a benchmark of bacterial isolates

We compared the performance of PlasClass to PlasFlow on the isolate assemblies from the benchmark in [6]. Specifically, we downloaded the assemblies and all bacterial and plasmid reference sequences used in the benchmarking experiment of [6] (available from: https://gitlab.com/sirarredondo/Plasmid_Assembly). Assembled contigs were mapped to the references using BLAST and contigs with matches (>95% mapping identity along >95% of the contig length) were assigned to the plasmid or chromosome class as described. There were 60579 contigs across all the assemblies of which 36172 matched one of the classes (8569 plasmid and 27603 chromosome) and were used in this test. As seen in Table 2, the majority of these sequences were extremely short (68% of the 36172 contigs <500bp). We looked at the impact of these short sequences by filtering out contigs below a certain length and the results of both methods improved when shorter sequences were filtered out. In all cases, PlasClass had consistently higher F1.

**Table 2:** Performance on bacterial isolates.

Contig length (bp)	# of contigs	PlasClass			PlasFlow		
		Precision	Recall	F1	Precision	Recall	F1
All	36172	43.65	77.58	55.87	31.16	87.77	46.00
>500	11659	53.15	91.30	67.18	37.68	89.23	52.99
>1000	7414	59.95	91.82	72.54	47.54	90.04	62.23
>5000	3999	61.84	92.12	74.00	50.05	92.31	64.91

Performance on bacterial isolates from [6], as a function of the minimum contig length.

### Performance on simulated metagenome assemblies

We simulated metagenomes by randomly selecting bacterial genome references from the NCBI along with their associated plasmids and using realistic distributions for genome abundance and plasmid copy number. For genome abundance we used the log-normal distribution, normalized so that the relative abundances sum to 1. For plasmid copy number we used a geometric distribution with parameter *p* = *min*(1, *log*(*L*)/7) where *L* is the plasmid length. This makes it much less likely for a long plasmid to have a copy number above 1, while shorter plasmids can have higher copy numbers. Short reads were simulated from the genome references using InSilicoSeq [7] and assembled.

We then classified the assembled contigs. Classification was performed on the assembled contigs that had a match to either a reference plasmid or reference chromosome sequence used in the simulation (1641 plasmid contigs, 32451 chromosome contigs in Sim1, and 14272 plasmid contigs, 374397 chromosome contigs in Sim2). F1 results are shown in Table 3. PlasClass outperformed PlasFlow by more than 17%. Scores were low for both methods due to the many short contigs in the assembly (50% and 73% of the contigs <500 bp in Sim1 and Sim2 respectively) and the class imbalance. We show the impact of short sequences on performance in Table 4. PlasClass consistently outperformed PlasFlow, and both methods performed better as more short sequences were filtered out.

**Table 3:** Performance on simulated metagenomes.

	# chromosomes	# plasmids	# unique	# contigs	PlasClass F1	PlasFlow F1
Sim1	34	82	56	34092	15.79	13.49
Sim2	198	333	219	388669	12.08	8.79

Summary of the simulated metagenome datasets and comparison of F1 scores. # unique is the number of distinct plasmids, ignoring multiple copies.

**Table 4:** Simulated metagenome performance by length.

	Contig length (bp)	# of contigs	PlasClass			PlasFlow		
			Precision	Recall	F1	Precision	Recall	F1
Sim1	All	34092	8.94	67.40	15.79	7.30	87.75	13.49
	>500	17023	11.22	78.55	19.64	8.20	85.05	14.95
	>1000	11696	15.67	80.96	26.26	10.92	85.00	19.36
	>5000	4032	36.11	86.80	51.00	28.09	90.80	42.91
Sim2	All	388669	6.64	66.98	12.08	4.64	84.31	8.79
	>500	106814	13.76	76.00	23.29	8.42	84.23	15.32
	>1000	45597	22.42	79.20	34.95	14.01	86.52	24.11
	>5000	5642	46.50	81.18	59.13	38.48	88.49	53.63

Performance on simulated metagenomes as a function of the minimum contig length.

### Performance on a plasmidome sample

We assembled the wastewater plasmidome sample ERR1538272 from the study by Shi et al. [8]. It is a metagenomic sample that was enriched for plasmid sequences. Each contig in the assembly was matched to the plasmid and bacterial reference databases using BLAST. The set of 9854 contigs (out of 35285) that matched the reference sequences (1888 plasmid contigs, 7966 chromosome contigs) was used as the gold standard to test the classifiers (contig length distribution is presented in S3 File See also S1 Fig). Although the plasmid-enriched setting favors PlasFlow, which sacrifices precision for higher recall, PlasClass still had a higher combined F1 as shown in Table 5.

**Table 5:** Performance on a plasmidome sample.

	Precision	Recall	F1 score
PlasClass	**32.32**	64.25	**43.01**
PlasFlow	23.72	**86.49**	37.23

Performance of PlasClass and PlasFlow on the plasmidome sample from [8].

We computed the precision-recall curve for the classification of the gold standard contigs in this sample by PlasClass, shown in S2 Fig (see also S3 File. The area under the curve is 0.41, more than double the baseline of 0.19 (the fraction of the contigs that are of plasmid origin).

### Classifying plasmids assembled from metagenomic samples

**Fig 1:**
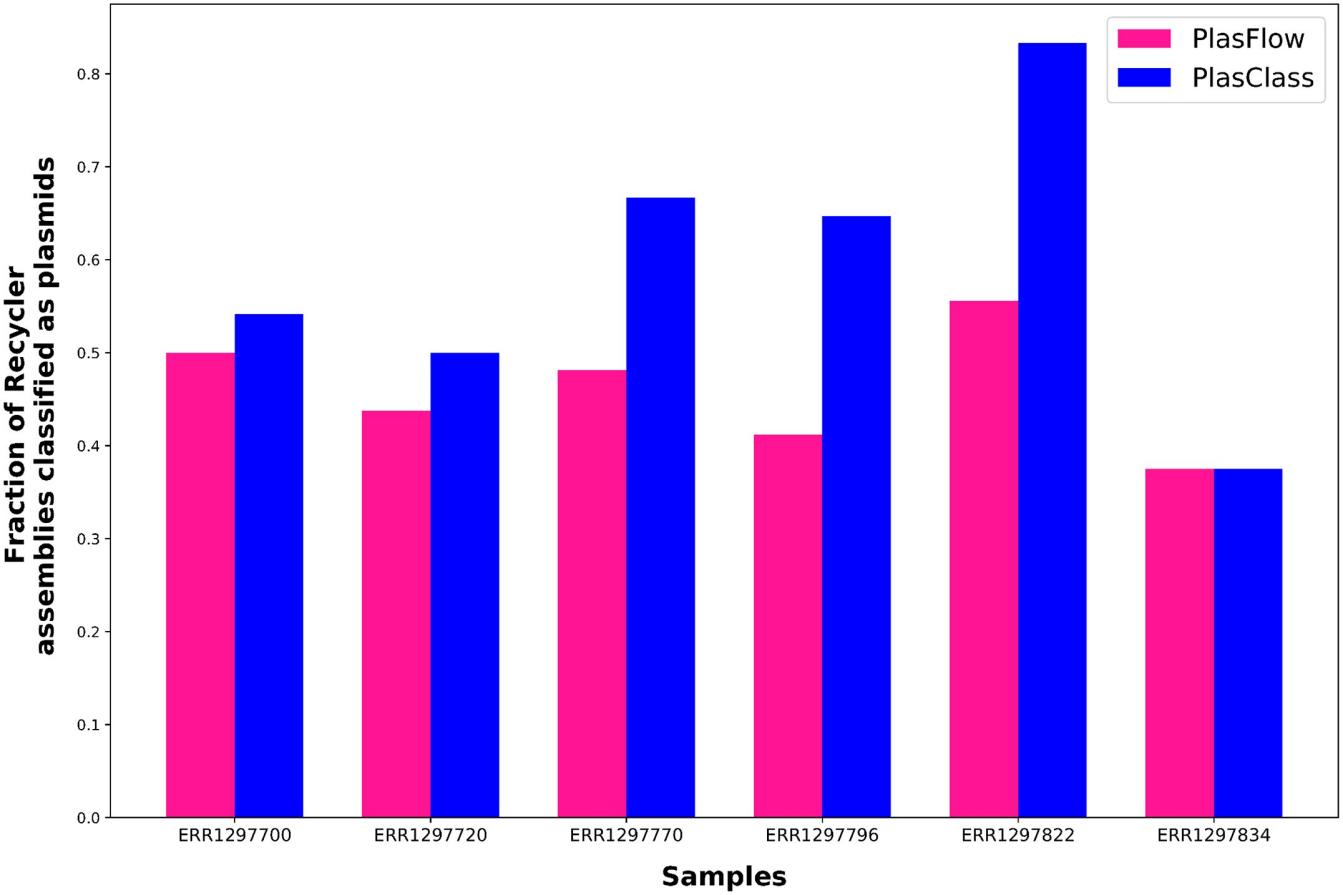
Classifying plasmids assembled from metagenomic samples. Agreement of PlasClass and PlasFlow classifications with the plasmids generated by Recycler.

We assembled six publicly available human gut microbiome samples (accessions: ERR1297700, ERR1297720, ERR1297770, ERR1297796, ERR1297822, ERR1297834) and found plasmid sequences in the assemblies using Recycler [9]. Recycler assembles plasmid sequences based on coverage and circularity—features that are not used by the classifiers. 16–27 plasmids were assembled per sample (median length: 3.4kb). We classified each of the plasmids generated by Recycler to determine the extent of agreement between the sequence classifiers and this orthogonal approach. As seen in Fig 1, PlasClass agreed with Recycler on the same number or more plasmids than PlasFlow in all samples. This suggests that PlasClass can correctly identify more plasmids in real datasets, which contain many previously unknown plasmid sequences.

### Resource usage

In Table 6, we compare the runtime and memory usage of PlasClass and PlasFlow on the full plasmidome, simulated metagenome, and isolate bacterial datasets. PlasClass (running with a single process) was faster than PlasFlow on the most time consuming sample and was significantly faster in all cases when using multiprocessing. It used less than half the RAM of PlasFlow and the RAM usage was not increased significantly when using multiprocessing. PlasFlow writes the feature matrices to disk while PlasClass does not. Performance was measured on a 44-core, 2.2 GHz server with 792 GB of RAM.

**Table 6:** Resource usage.

Dataset	PlasFlow			PlasClass		PlasClass—8 processes	
	Runtime	RAM	Disk	Runtime	RAM	Runtime	RAM
Isolates	12.8	47.8	21.4	36.3	17.2	6.8	17.2
Sim1	7.1	28.3	12.1	16.2	12.0	3.0	12.0
Sim2	89.3	291.3	137.5	54.8	17.3	17.1	17.3
Plasmidome	7.9	28.8	12.2	4.2	12.2	5.2	17.3

Runtime (wall clock time, in minutes) and memory usage (in GB) of PlasClass and PlasFlow.

## Discussion

We presented the PlasClass algorithm for classifying plasmid sequences. We applied the algorithm across a wide range of contexts and showed that in most cases PlasClass outperformed the state-of the-art algorithm PlasFlow. It was also faster and required less memory.

The task of classifying plasmid sequences in the real-world context of metagenomic data is a difficult task due to the nature of the assembled sequences: the sequences are mostly short (60-90% are shorter than 1 kbp, see Tables 2 and 4), and there is an imbalance between the number of plasmid and bacterial sequences (1:3 in the bacterial isolates, and 1:4 in the plasmid-enriched plasmidome samples presented). Given the constraints, the quality of classification is naturally limited, but the task is of high importance for understanding plasmid role in horizontal transfer, antibiotic resistance and ecology. We also showed that classification quality improves when focusing on longer sequences and when plasmid sequences are enriched.

## Availability and future directions

PlasClass is open-source and freely available under the MIT license. PlasClass is maintained on GitHub, enabling bug-reporting and community collaboration in extending the tool to meet needs of the users as they arise. It can be found at https://github.com/Shamir-Lab/PlasClass.

We plan to use PlasClass in order to improve plasmid assembly from metagenomic samples, by utilizing the classification scores of contigs. Another possible future direction is to tailor the plasmid training data to the problem at hand: Currently we use all known plasmids for training, which creates a bias towards clinically relevant samples. By using training datasets tailored to other specific environments one can create a classifier that would fit those environments better.

## Supporting information

S1 File PlasClass documentation. Complete documentation for using PlasClass.
(PDF)

S2 File Results with ambiguous sequences filtered. Extended results reporting performance with ambiguous sequences filtered out.
(PDF)

S3 File Plasmidome dataset extended results. Extended results reporting the contig lengths and precision-recall curve for the plasmidome sample.
(PDF)

S1 Fig Plasmidome contig lengths. Histogram of the contig lengths in the plasmidome assembly. Note that the y-axis uses log-scale.
(TIF)

S2 Fig Plasmidome precision-recall curve. Precision-recall curve for the classification of contigs of in the plasmidome sample.
(TIF)
