# Supplementary material for: PlasClass improves plasmid sequence classification
Source: PLoS Comput Biol. 2020 Apr 3;16(4):e1007781. doi: 10.1371/journal.pcbi.1007781 (PMC7159247; doi:10.1371/journal.pcbi.1007781)
Supplement: S1 File — Complete documentation for using PlasClass. (PDF) [file pcbi.1007781.s001.pdf]

Supplement S1 - Supplementary information for:
PlasClass improves plasmid sequence classification

S1 PlasClass documentation

PlasClass is fully documented at `https://github.com/Shamir-Lab/PlasClass`. We outline the installation and usage instructions here.

Installing PlasClass

PlasClass is written in Python3 and requires NumPy and scikit-learn. To install PlasClass do:

```
git clone https://github.com/Shamir-Lab/PlasClass.git
cd PlasClass
python setup.py install
```

We recommend using a virtual environment.

After installation, run the tests to see that PlasClass is installed and working correctly on your system:

```
python test/test.py
```

Classifying sequences in a fasta file

The `classify_fasta.py` script can be used to classify the sequences in a fasta file. It is invoked as follows:

```
python classify_fasta.py -f <input file> [-o <output file>] [-p <# processes>]
```

The required option is:

- `-f/--fasta`: String - the fasta file to be classified.

The two optional options are:

- `-o/--outfile`: String - the name of the output file.
  Default: `<in>.probs.out` (`<in>` is the path to the fasta file).
- `-p/--num_processes`: Integer - the number of processes to use. Default: 8.

The output file is a tab separated file with each line containing a sequence header and the corresponding score. The sequences are in the same order as in the input fasta file.

Calling the PlasClass module

The classifier can also be imported and called directly in a Python program. For example, once the `plasclass` module has been installed the following lines of code can be used:

```
from plasclass import plasclass
my_classifier = plasclass()
my_classifier.classify(seqs)
```

The `plasclass()` constructor takes 3 optional parameters:

- `n_procs`: Integer - Number of processes to use (default: `1`)
- `scales`: Integer array - Scales of the sequence length bins (default: `[1000,10000,100000,500000]`)
- `ks`: Integer array - Values of the k-mer lengths to use (default: `[3,4,5,6,7]`)

The sequence(s) to classify, `seqs`, can be either a single string or a list of strings. The strings must be uppercase. The function `plasclass.classify(seqs)` returns a list of plasmid scores, one per input sequence, in the same order as the input.

Training PlasClass models

The user can train models by providing two fasta files with plasmid and bacterial reference sequences to the `train.py` script. Required options are:

- `-p/--plasmid`: String - the fasta file of the plasmid references.
- `-c/--chromosome`: String - the fasta file of the chromosome references.
- `-o/--outdir`: String - the path of the output directory.
  Default: directory of script.

The optional options are:

- `-n/--num_processes`: Integer - the number of processes to use.
  Default: 16.
- `-k/--kmers`: String - comma separated list of the k-mer sizes to use.
  Default: 3,4,5,6,7.
- `-l/--lengths`: String - comma separated list of the sequence lengths to use.
  Default: 1000,10000,100000,500000.

The models will be created in the output directory and should be put into the `data` directory of the PlasClass module to be used. Note that if k-mer and sequence lengths other than the default are used, then these must be specified when calling the `plasclass()` constructor.
