# Supplementary material for: PlasClass improves plasmid sequence classification
Source: PLoS Comput Biol. 2020 Apr 3;16(4):e1007781. doi: 10.1371/journal.pcbi.1007781 (PMC7159247; doi:10.1371/journal.pcbi.1007781)
Supplement: S2 File — Extended results reporting performance with ambiguous sequences filtered out. (PDF) [file pcbi.1007781.s002.pdf]

Supplement S2 - Supplementary information for:
PlasClass improves plasmid sequence classification

S2 Filtering out ambiguous sequences

We calculated performance of PlasClass and PlasFlow on assembled contigs when considering any sequence that matches a plasmid reference to belong to the plasmid class as was done in other works [1, 2]. A more conservative construction of the gold-standard set of contigs to use when benchmarking a sequence classifier would remove these sequences. Here we present key results from Tables 2, 3, and 5, recalculated using the more strict condition that sequences that match both plasmid and bacterial references are excluded. In that setting every sequence uniquely matches either plasmid or the bacterial references but not both. Results are reported in Table S1.

Table S1. Performance with ambiguous matches filtered out.

Sample	# plasmid contigs	# bacterial contigs	PlasFlow Precision	PlasFlow Recall	PlasFlow F1	PlasClass Precision	PlasClass Recall	PlasClass F1
Isolates	74	27603	0.401	90.54	0.799	0.751	87.84	1.49
Sim1	1120	32451	5.13	88.21	9.69	6.73	72.59	12.32
Sim2	11579	374397	3.82	84.93	7.31	5.83	71.97	10.79
Plasmidome	1152	7966	16.36	89.15	27.61	22.81	65.19	33.81

Performance calculated when ambiguous matches to both plasmid and bacterial references are filtered out.

In all cases PlasClass outperformed PlasFlow, and by a similar factor as when using the less strict procedure reported in the Results. The scores of both methods are much lower than when ambiguous sequences are not filtered out since the stricter filtering creates a much larger imbalance between the number of the plasmid and bacterial contigs as seen in Table S1 (for example, 1:500 in the bacterial isolates). To see why this is the case consider, an assembly with 10,000 contigs, of which 100 are of plasmid origin. A random baseline, which predicts that a sequence is of plasmid origin with 50% probability, will correctly classify 50 of the plasmid contigs on average, achieving a recall of 50%. However, it will also classify on average half of the bacterial contigs as plasmids, achieving a precision of only 1% and an F1 score of 2%. An alternative random baseline using the correct 1% probability for plasmid sequences would achieve precision, recall and F1 of 1%. So one would expect substantially lower F1 scores than

in more balanced classification setups.

As in the Results section, filtering out shorter sequences improved both of the methods, and PlasClass consistently outperformed PlasFlow by a similar factor (results not shown). This validates the main findings that, even using this more strict procedure, PlasClass does better than PlasFlow across a wide range of contexts.

References

1. Krawczyk PS, Lipinski L, Dziembowski A. PlasFlow: predicting plasmid sequences in metagenomic data using genome signatures. Nucleic Acids Research. 2018;46(6):e35.
2. Arredondo-Alonso S, Willems RJ, van Schaik W, Schürch AC. On the (im)possibility of reconstructing plasmids from whole-genome short-read sequencing data. Microbial genomics. 2017;3(10):e000128.
