# Supplementary material for: PlasClass improves plasmid sequence classification
Source: PLoS Comput Biol. 2020 Apr 3;16(4):e1007781. doi: 10.1371/journal.pcbi.1007781 (PMC7159247; doi:10.1371/journal.pcbi.1007781)
Supplement: S3 File — Extended results reporting the contig lengths and precision-recall curve for the plasmidome sample. (PDF) [file pcbi.1007781.s003.pdf]

## Supplement S3 - Supplementary information for: PlasClass improves plasmid sequence classification

# S3 Plasmidome sample

Fig S1 presents the distribution of contig lengths in the assembly graph of the wastewater plasmidome sample ERR1538272 from the study by Shi et al. [1].

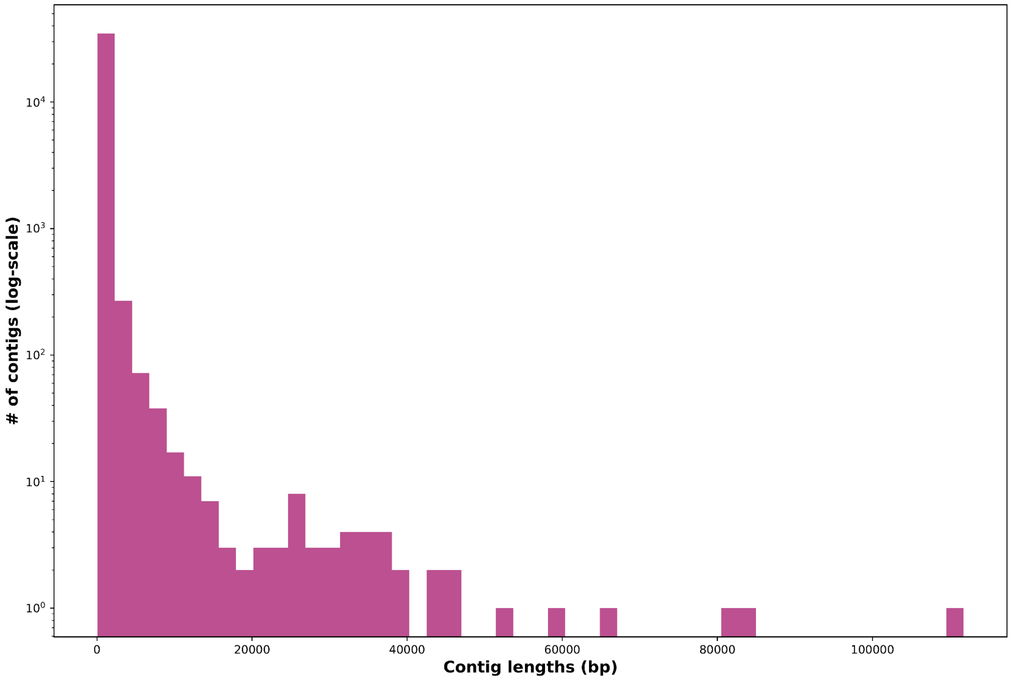


**Fig S1. Contig lengths.** Histogram of the contig lengths in the plasmidome assembly. Note that the y-axis uses log-scale.

Fig S2 presents the precision-recall curve for the classification of the plasmidome sample by PlasClass. The area under the precision-recall curve is AUPR=0.41. The baseline given the class imbalance is 0.19.

# References


1. Shi Y, Zhang H, Tian Z, Yang M, Zhang Y. Characteristics of ARG-carrying plasmidome in the cultivable microbial community from wastewater treatment system under high oxytetracycline concentration. Applied microbiology and biotechnology. 2018;102(4):1847–1858.

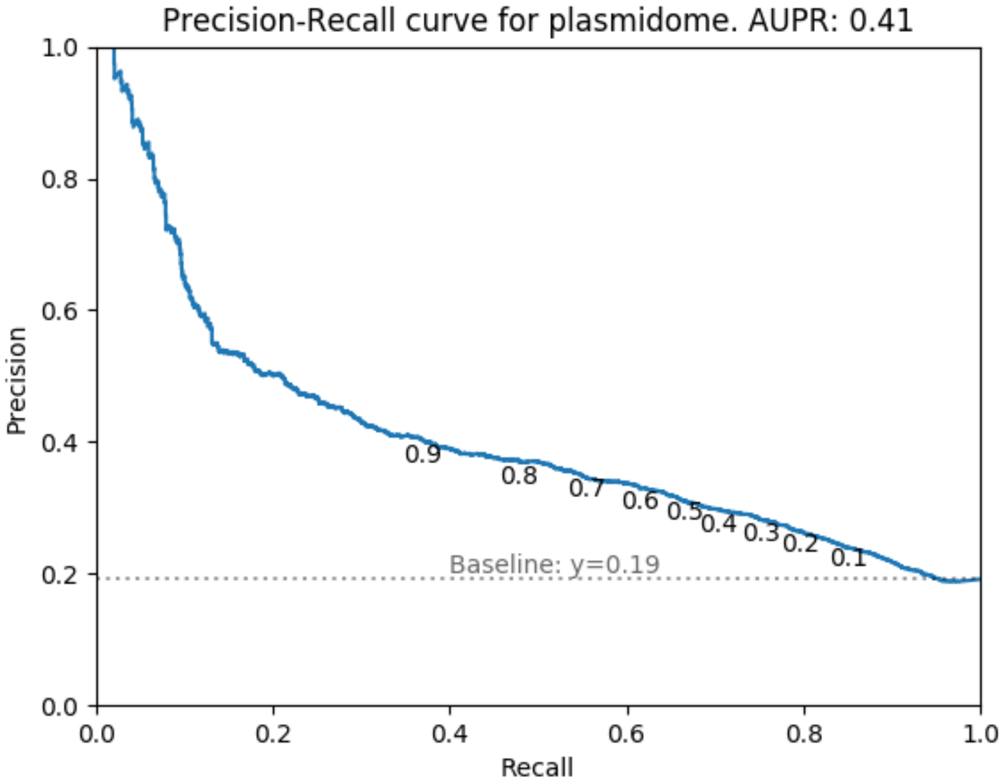


**Fig S2. Precision-recall curve.** Precision-recall curve for the classification of contigs of in the plasmidome sample.
