# Supplementary figures and images for: PlasClass improves plasmid sequence classification
Source: PLoS Comput Biol. 2020 Apr 3;16(4):e1007781. doi: 10.1371/journal.pcbi.1007781 (PMC7159247; doi:10.1371/journal.pcbi.1007781)

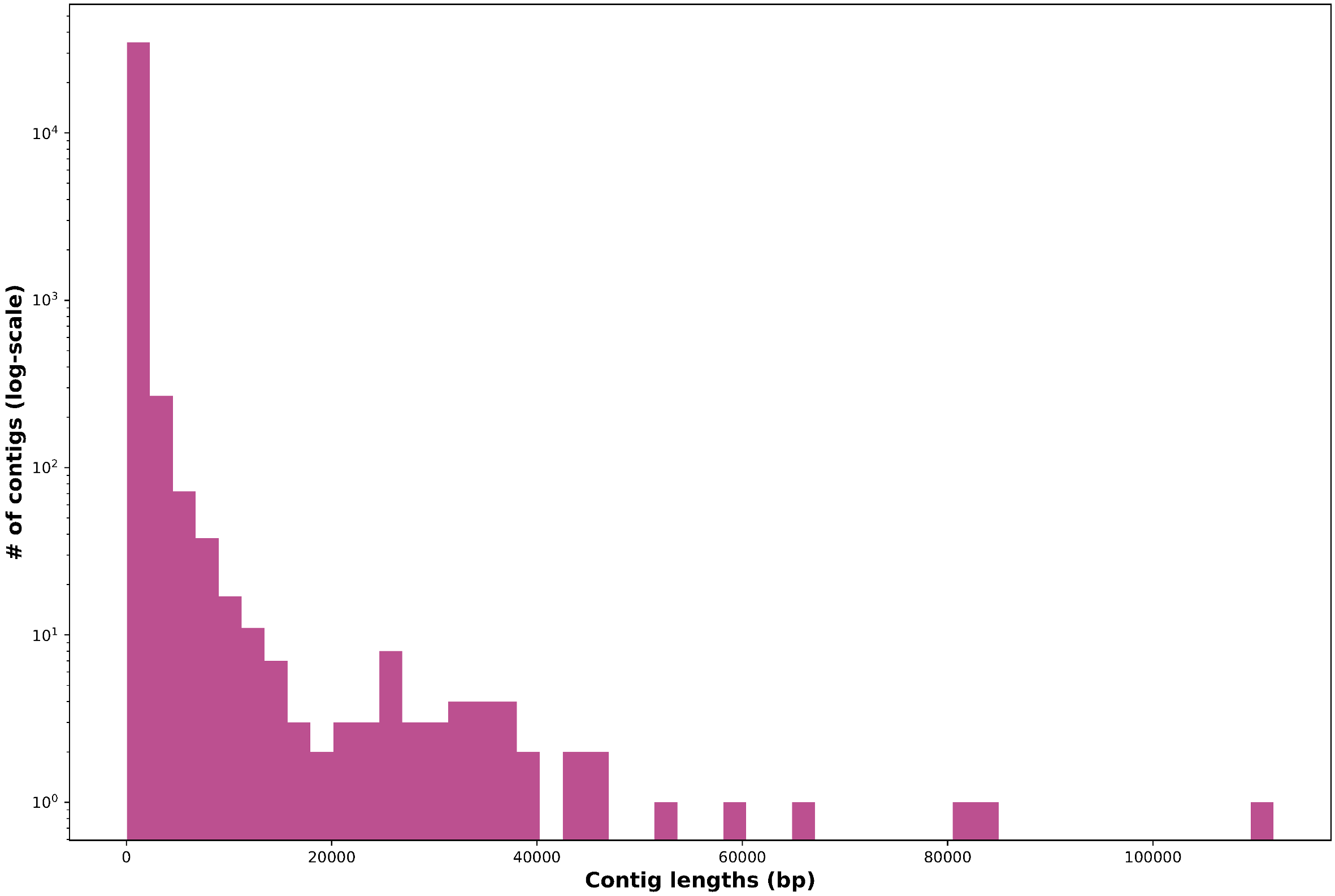

Supplement: S1 Fig — Histogram of the contig lengths in the plasmidome assembly. Note that the y-axis uses log-scale. (TIF) [file pcbi.1007781.s004.tif]

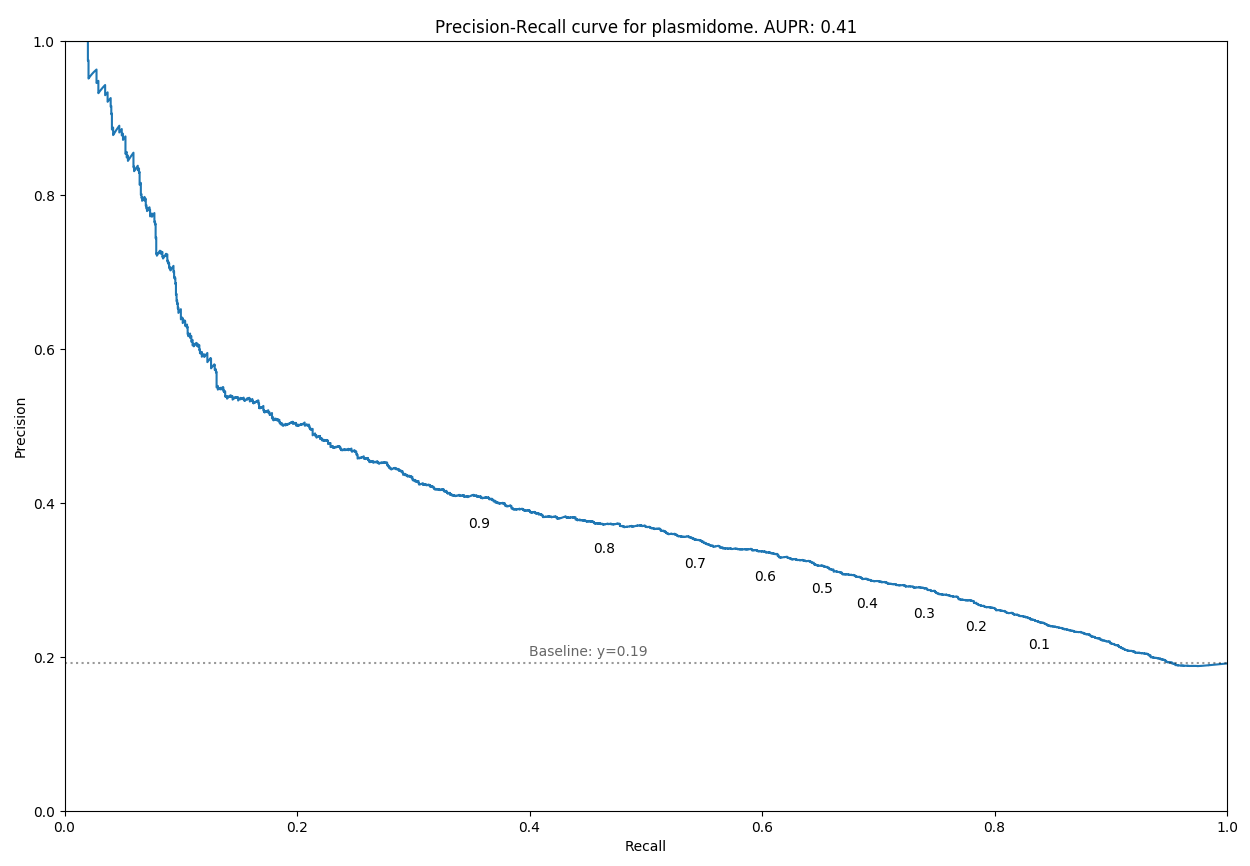

Supplement: S2 Fig — Precision-recall curve for the classification of contigs of in the plasmidome sample. (TIF) [file pcbi.1007781.s005.tif]
